# Induction of Defense-Related Enzymes in Banana Plants: Effect of Live and Dead Pathogenic Strain of *Fusarium oxysporum* f. sp. *cubense*

**DOI:** 10.5402/2013/601303

**Published:** 2012-12-06

**Authors:** Janki N. Thakker, Samiksha Patel, Pinakin C. Dhandhukia

**Affiliations:** ^1^Department of Biotechnology, PD Patel Institute of Applied Sciences, Charotar University of Science and Technology, CARUSAT Campus, Gujarat, Changa 388421, India; ^2^Department of Integrated Biotechnology, Ashok and Rita Patel Institute of Integrated Study and Research in Biotechnology and Allied Sciences, Gujarat, New Vallabh Vidyanagar 388 121, India

## Abstract

The aim of the present study was to scrutinize the response of banana (Grand Naine variety) plants when interacting with dead or live pathogen, *Fusarium oxysporum* f.sp. *cubense*, a causative agent of Panama disease. Response of plants was evaluated in terms of induction of defense-related marker enzyme activity, namely, peroxidase (POX), polyphenol oxidase (PPO), *β*-1,3 glucanase, chitinase, and phenolics. Plant's interaction with live pathogen resulted in early induction of defense to restrain penetration as well as antimicrobial productions. However, pathogen overcame the defense of plant and caused disease. Interaction with dead pathogen resulted in escalating defense response in plants. Later on plants inoculated with dead pathogen showed resistance to even forced inoculation of live pathogen. Results obtained in the present study suggest that dead pathogen was able to mount defense response in plants and provide resistance to Panama disease upon subsequent exposure. Therefore, preparation from dead pathogen could be a potential candidate as a biocontrol agent or plant vaccine to combat Panama disease.

## 1. Introduction

*Musa acuminata* (Banana) is one of the most important fruit crops of world as well as of India. Banana could be considered poor man's apple, and it is the cheapest among all other fruits in the country. Fusarium wilt caused by *Fusarium oxysporum *f.sp. *cubense* (Foc) is the most destructive disease of banana [1]. The pathogen is soil-borne and remains viable up to several years and cause 20%–80% loss of banana. Several disease management strategies can be used such as crop rotation, burning infected plants or plant parts, and application of carbendazim [2]. Methods mentioned have limited success, and the application of synthetic fungicides may result in undesirable effects on the environment. 

An alternative to above strategies for managing fusarium wilt is the use of biological control. Biocontrol agent can be a beneficial organism (live or dead) or its part such as cell wall, protein, and oligosaccharides [3]. While using live organisms as a biocontrol agent, appropriate conditions for maintaining it should be strictly followed. Nevertheless, if part of the organism such as cell wall, protein, oligosaccharide, or attenuated/killed organism is used then strict conditions are not required. Plants, humans, and animals give instantaneous response to the pathogen or its part. Animals and humans produce antibodies against pathogen or vaccine, similarly plants response to pathogen attack by producing PR-proteins, defense-related enzymes [4], plantibodies, and phytoalexins. 

Study of PR-proteins or defense-related enzymes are key to any plant disease resistance mechanism. Farmers in Gujarat, India purchase tissue culture plants every year for sowing in the fields with the expectations of getting high production and high profit. Foc being soil borne may enter the plant and cause disease anytime after sowing and affects fruit production. If plants are immunized, that is, accumulation of defense-related enzymes occurs before the attack of pathogen then the pathogen can be successfully warded off, and loss can be minimized. Same concept of vaccination is used here. Vaccines used for animals and humans are derived from the same disease causing organism. But vaccines have inactive organism or attenuated organism. Elicitor used here is acting as a vaccine (derived from the dead fungus) to protect the plant. The aim of the present study was to compare the interaction of dead and live pathogen with banana plants (Grand Naine variety). Grand Naine is a large fruit yielding dwarf Cavendish variety with height of 6.5 to 7.5 ft introduced to India from Israel remains choice of farmers as the bunches of banana fruits can be harvested within twelve to thirteen months from the date of planting. The plant response was documented using several marker enzymes to analyze whether the plant could differentiate the signals from dead and live pathogen. Another important aim was to check if the response generated using dead pathogen was sufficient to ward off the forced inoculated live pathogen. 

## 2. Materials and Methods 

### 2.1. Maintenance of Fungal Culture

Previously isolated culture of *Fusarium oxysporum* f.sp. *cubense* (Foc) from fusarial wilt infected banana plants was maintained on potato dextrose agar (PDA) at 27°C [4]. For liquid culture of fungus, 8-mm agar plug of 3-4 w k old culture was inoculated in potato dextrose broth (PDB) and incubated at 27°C for 21 day on PDB. The media with mycelium was autoclaved at 121°C for 20 minutes, crushed intensely in grinder, and was used as dead fungi for treatment.

#### 2.1.1. Plant Material

Two months old Banana (Grand Naine variety) plantlets were procured from Gujarat State Fertilizers and Chemicals (GSFC), Baroda, Gujarat. Plants were planted and maintained in campus garden. Health of these plants was observed regularly by visual inspection.

### 2.2. Dead and Live Fungus Treatment

Plant roots of Grand Naine variety were exposed without damaging them by carefully removing the surrounding soil. Dead and live fungus suspensions were prepared by mixing 1 g of dead fungus and live fungus per liter of distilled water. Per plant, 1 mL of dead and live pathogen suspension was administered against control plants treated with 1 mL of distilled water in exposed root region. After treatment, changes in levels of defense-related enzymes in leaves were assayed after each successive day till seventh day [4, 5].

### 2.3. Experimental Design

Disease-free in vitro propagated two months old plantlets were selected. Dead and live fungus treatments were given as mentioned earlier to plants. Distilled water treated plants were used as control. Leaves were excised up to seven days at regular interval of 24 h for estimation of defense related enzymes, namely, POX, PPO, *β*-1,3 glucanase, chitinase, and total phenolics from both control and treated plants. Experiments were repeated twice under similar conditions with different sets of plants, and all the analyses were performed in triplicates. Fresh Banana leaves were washed in running tap water and homogenized in liquid nitrogen. The homogenized leaves were kept at 4°C until used for enzyme analyses. POX activity was measured as described by Sadashivam and Manickam [6]. PPO, *β*-1,3 glucanase, chitinase, and total phenolics assays were done as described by Meena et al. [7].

### 2.4. Forced Inoculation

To ensure the induction of resistance, plant roots treated with dead pathogen was exposed to spore suspension of Foc (10^4^ spores/mL) where plants treated with distil water was used as control and exposed to same quantity of spore suspension as experimental. Plants were kept under observation for the development of the symptoms. 

## 3. Results 

The aim of this study was to investigate interaction of dead and live pathogen, *Fusarium oxysporum* f.sp. *cubense*—a causative agent of panama disease, with banana plants (Grand Naine). Interaction was determined using accumulation of several defense related enzymes, namely, POX, PPO, *β*-1,3 glucanase, chitinase, and total phenolics as markers for induction of defense. Hypothesis was that plants could differentiate signals from dead and live pathogen and in response to signals from dead pathogen, it can induce defense enzymes, which could protect plants upon subsequent exposure to pathogen. 

### 3.1. Effect of Live and Dead Pathogen on Defense-Related Enzymes

**Figure 1:**
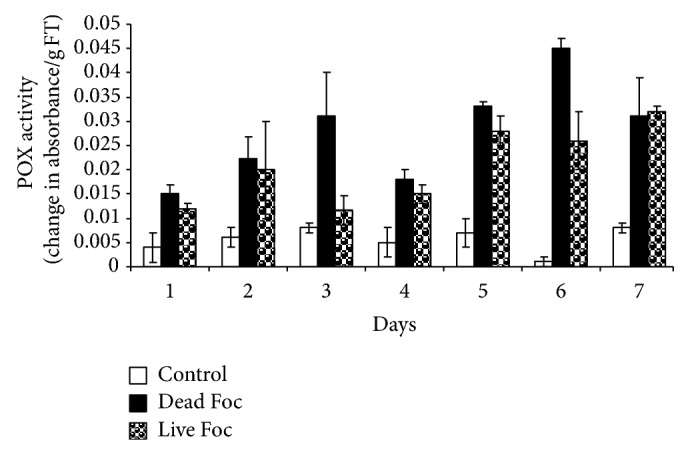
POX activity profile of banana for reinforcement of physical barrier as well as ROS generation in response to distilled water (control), live fungus, and dead fungus interactions for seven days.

**Figure 2:**
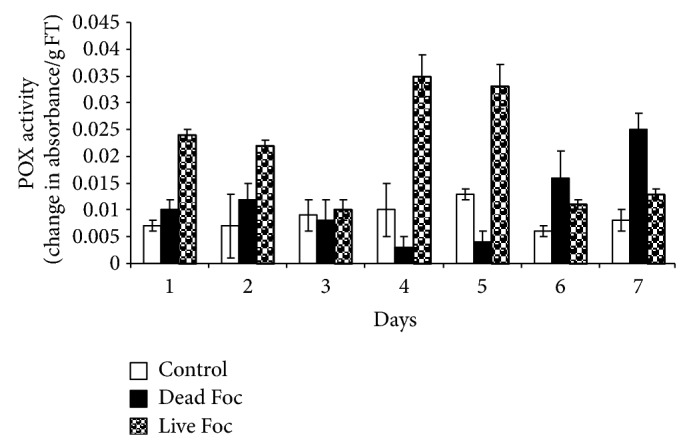
PPO activity profile of banana for generation of antimicrobials in response to distilled water (control), live fungus, and dead fungus interactions for seven days.

POX activity in control plants remained constant throughout seven days of study. POX activity induced earlier in dead and live pathogen treated plants and retained elevated levels compared to control plants. POX activity increased 4- and 2.5-fold in dead and live fungus treated plants, respectively. Highest POX activity in plant treated with dead and live pathogen was observed on 6th and 7th day, respectively (Figure 1). Comprehensible difference in PPO activity was observed on treatment of dead and live pathogen. With live fungus interaction, plants showed to induce 3-fold PPO activity from first day onward, reached highest level on 4th day, and thereafter reached near basal. However, dead fungus treatment failed to mount significant induction in PPO activity for initial five days of interaction followed by 2- and 3-fold induction in PPO activity on 6th and 7th days, respectively compared to control plants (Figure 2).

**Figure 3:**
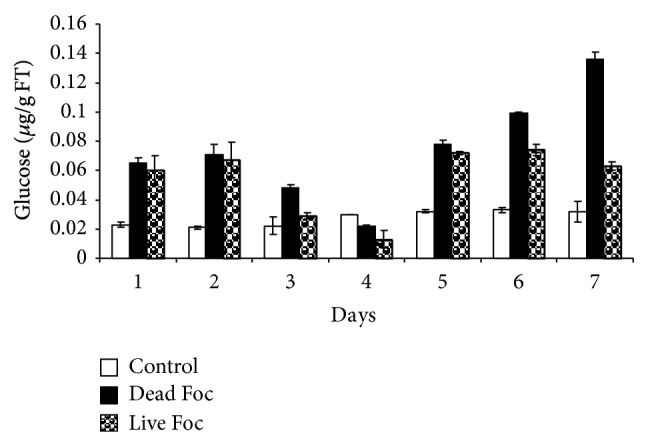
*β*-1,3 glucanase activity profile of bananafor direct antifungal activity in response to distilled water (control), live fungus, and dead fungus interactions for seven days.

Interaction of dead and live fungus with banana plants resulted in induction of *β*-1,3 glucanase activities from 1st day onward. Up to five days, pattern of *β*-1,3 glucanase activity was very similar in both treatments. From the 5th day onward, *β*-1,3 glucanase activities stabilized at 2- to 2.5- fold higher levels in live fungus interaction as compared to control. In case of dead fungus interaction, gradual increase in *β*-1,3 glucanase activity was observed from 5th day onward with highest increase 3.5-fold on 7th day as compared to control (Figure 3).

**Figure 4:**
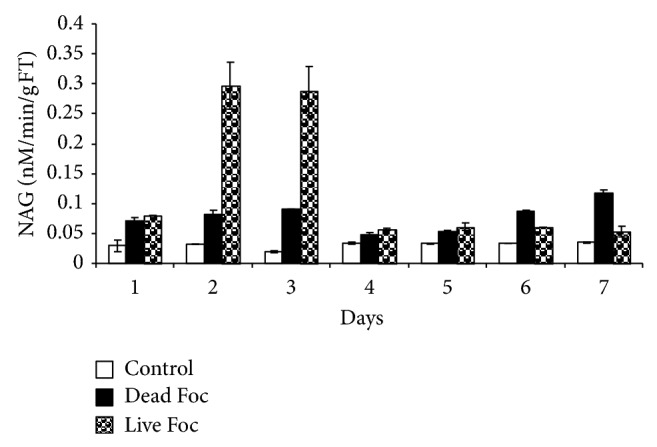
Chitinase activity profile of banana for direct antifungal activity in response to distilled water (control), live fungus, and dead fungus interactions for seven days.

Chitinase activity induced from 1st day in banana plants treated with dead and live pathogenic fungus.  Figure 4 showed stiff rise (10-fold) in chitinase activity on 2nd and 3rd day followed by rapid fall and retention of about 1.5 fold chitinase activity in banana treated with live fungus compared to control plants. Whereas, in dead fungus treated plants, chitinase activity increased twofold on 1st day followed by gradual increase up to 4-fold on 7th day. 

**Figure 5:**
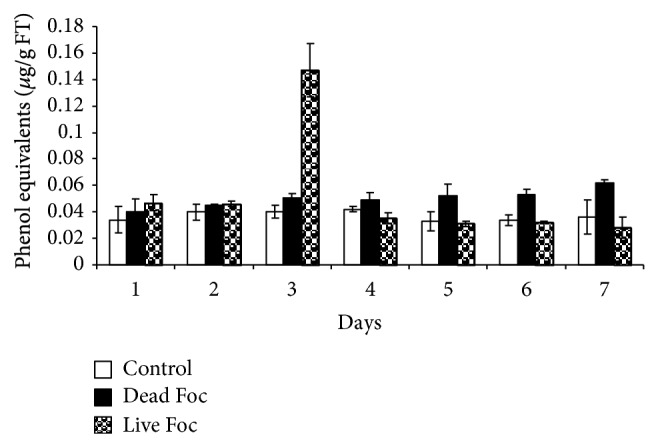
Total phenolics activity profile of banana for reinforcement of physical barrier as well as antimicrobial activity in response to distilled water (control), live fungus, and dead fungus interactions for seven days.

In dead fungus treated plants, total phenolics content gradually increased and reached to maximum on 7th day (2-fold). Whereas in live fungus treated plants, total phenolics content increased on 3rd day followed by sharp decline to reach basal level as compared to control plants (Figure 5).

### 3.2. Forced Inoculation

Control plants and plants treated with dead pathogen were forced inoculated with the live fungal spores and observed for the development of symptoms. In control plants, the characteristic symptoms of fusarial wilt were observed in the first week after forced inoculation followed by aggravation of disease condition after 15 days. However, no symptoms of fusarium wilt were observed even after two months after forced inoculation in plants treated with dead pathogen.

## 4. Discussion 

In the present study, analysis of plants response towards dead and live pathogenic strain was carried out using induction of several key marker enzymes associated with plant defense mechanism. Development of effective, durable, economic, and environmentally sound strategies for the control of crop diseases could be possible through an improved understanding of the interactions between plants and pathogenic agents. The ability of a pathogen to produce a disease in a host plant is usually the exception, not the rule. This is because plants have an innate ability to recognize potential invading pathogens and to set up successful defenses. On the other hand, successful pathogens produce diseases because they are capable to evade detection or suppress host defense mechanisms, or both [8]. 

When plants are challenged by a pathogen, early local defense reactions and delayed, systemic responses get activated in order to counteract the pathogen attack. Among the early local responses, the hypersensitive response (HR) leads to a local programmed cell death in order to deprive the pathogens of their nutrition base [9]. Later on, the plant can develop systemic acquired resistance (SAR) leading to resistance throughout the whole plant in an unspecific manner towards a broad spectrum of pathogens. In case of SAR, signal is transmitted from infected tissue in the whole plant for induction of overall defense gene expression. This demonstrates that signal perception in initial pathogen recognition and signal transduction to initiate further defense responses is essential for plants to counteract phytopathogens [10].

Some defenses are constitutive, such as various pre-formed antimicrobial compounds, whereas others activated by pathogen recognition. Recognition process includes product of a dominant or semidominant resistance R gene present in the plant and the corresponding dominant avirulence (Avr) factor encoded by or derived from the pathogen. Recognition of Avr factor by host plant starts one or more signal transduction pathways that activate several of plant's defenses, thus compromising ability of pathogen to colonize plant [11, 12]. 

In the present study, interaction of banana plants with dead and live pathogen resulted in induction of POX activity. However, POX activity induced more with dead fungus. As dead pathogen was capable to generate initial recognition signals; however, it cannot counter the plant response, which leads to induction of POX activity more than live fungus. Peroxidases are a well-known class of PR proteins and induced in host plant tissues by pathogen infection. They belong to PR-protein 9 subfamily [13] and are expressed to limit cellular spreading of infection through establishment of structural barriers or generation of highly toxic environments by massively producing ROS and RNS [14]. Peroxidase activity or Peroxidase gene expression in higher plants is, indeed, induced by fungi [15], bacteria [16], viruses [17], and viroids [18]. Cross-linking of the phenolic monomers in oxidative coupling of lignin subunits has been associated with peroxidase using H_2_O_2_ as oxidant. Acidic and basic peroxidases are capable of oxidizing p-coumaryl and coniferyl alcohol. One significant event in plant defense reactions is oxidative burst, a common early response of host plant cells to pathogen attack and elicitor treatment. Our results showed increase in POX activity in dead fungus treated as well as fungus treated plants as compared to control. This result indicates that elicitor slowly increases level of lignin formation, suberization, and hypersensitive response. Similar results were reported in wheat heads [19]. 

Other enzymes, PPOs are a group of copper containing enzymes that catalyze oxidation of hydroxy phenols to their quinone derivatives, which have antimicrobial activity [20]. Because of its reaction products and wound inducibility, PPO plays a role in defense against plant pathogens [21]. Plant immediately respond to pathogen so there is immediate rise in PPO indicating immediate synthesis of antimicrobials to ward off pathogen. In elicitor treated plant PPO activity increases slowly day by day indicating that plant has got stimuli to increase PPO. In case of live pathogen interaction, there was immediate response by plants increasing PPO 5-fold on the first day, which starts decreasing from the 6th day indicating multiplication of fungus in the plant system. However, dead pathogen completely fails to mount this response, which strongly suggests that plants are capable to differentiate signals from live and dead pathogen. Increase in PPO activity was reported in banana roots treated with Foc-derived elicitors by Thakker et al. [4]. Marked increase in PPO activity was observed in banana roots treated with *Psuedomonas fluorescens* against fusarial wilt [22].

*β*-1,3 glucan and chitin, polymer of N-acetylglucosamine (NAG) are major cell wall components of many fungi. Since *β*-1,3 glucanase and chitinases have been shown to be capable of attacking cell wall of fungal pathogens, these enzymes have been proposed as direct defense enzymes of plants [23]. We observed an increase in chitinase and *β*-1,3 glucanase activity indicating plants ready mechanism to ward off pathogen by directly degrading the pathogen cell wall and in turn protecting the plant. In our previous study, we found antifusaric activity of elicitor induced *β*-1,3 glucanase that showed swelling of mycelia after one hour incubation of pathogen with purified elicitor [24]. Endochitinase purified from barley was capable of inhibiting the growth of *Trichoderma rescei*, *Alternaria alternate* and *Neurospora crassa* [25]. In addition, Mauch et al. [26] reported that in combination, chitinase and *β*-1,3 glucanase act synergistically to inhibit fungal growth.

Phenolic acids are involved in phytoalexin accumulation, biosynthesis of lignin, and formation of structural barrier, and play a main role in the resistance against pathogen. Marked accumulation of phenolics was observed on the 3rd third day in fungus treated plants indicating the plants sensitivity to pathogen, and it attempt to protect itself by the formation of structural barriers. Further, from the 4th day activity decreases showing successful multiplication and establishment of pathogen by overcoming structural barriers formed by plants. In dead pathogen-treated plants, we found slow increase in phenolics activity indicating ability to recognize dead pathogen as some foreign body, but it is not multiplying; therefore, it will not break structural barrier. Marked accumulation of phenols leading to suppression of Fusarial wilt was observed in tomato plants [27]. Similar results were observed in banana plants *in vitro* condition [4]. When tomato plants were treated with catechol, marked accumulation of phenols was observed and it resulted in suppression of *Fusarium* wilt of tomato [27]. Anna and Dubery [28] investigated that subfraction of cell-wall bound phenolics, ester-bound phenolics, glycoside bound phenolics, and free phenolics increased 6.3-, 4.2-, 3.0-, and 2.3-folds, respectively, upon induction.

In plants treated with dead pathogen, a forced inoculation of live pathogen was performed to assess induced plant's ability to ward off pathogen. Interestingly no symptoms of fusarial wilt were observed in these plants even though pathogen was in close proximity. This supports the view that elicited plants are less susceptible to infection. 

## 5. Conclusion

The present study strongly supports the view of preparation of plant vaccines for combating devastating disease like Fusarium wilt of banana. Dead pathogen preparation was not only successful in mounting defense response but also in protecting plants upon subsequent infections. Therefore, it could be potential candidate for plant vaccine preparation to combat panama disease.

## References

[1] Moore N. Y., Pegg K. G., Bentley S., Smith L. J., Molina A. B., Nikmasdek N. H., Liew K. W. (2001). Fusarium wilt of banana: global problems and perspectives. *Banana Fusarium Wilt Management: Towards Sustainable Cultivation*.

[2] Thangavelu R. A., Palaniswami B., Velazhahan R. (2004). Mass production of *Trichoderma harzianum* for managing fusarium wilt of banana. *Agriculture Ecosystem Environment*.

[3] Boukaew S., Chuenchit S., Petcharat V. (2011). Evaluation of Streptomyces spp. for biological control of Sclerotium root and stem rot and Ralstonia wilt of chili pepper. *BioControl*.

[4] Thakker J. N., Patel N., Kothari I. L. (2007). *Fusarium oxysporum* derived Elicitor-induced changes in Enzymes of Banana leaves against wilt disease. *Journal of Mycology Plant Pathology*.

[5] Thakker J. N., Patel P., Dhandhukia P. C. (2011). Induction of defense-related enzymesin susceptible variety of banana: role of Fusarium derived elicitors. *Achieves of Phytopathology and Plant Protection*.

[6] Sadashivam S., Manickam A. (1992). *Enzymes: Biochemical Methods*.

[7] Meena B., Marimuthu T., Velazhahan R. (2001). Salicylic acid induces systemic resistance in groundnut against late leaf spot caused by *Cercosporidium personatum*. *Journal of Mycology and Plant Pathology*.

[8] Borrás-Hidalgo O. (2004). Basic insight in plant-pathogen interaction. *Biotecnologia Aplicada*.

[9] Greenberg J. T., Vinatzer B. A. (2003). Identifying type III effectors of plant pathogens and analyzing their interaction with plant cells. *Current Opinion in Microbiology*.

[10] Nürnberger T., Scheel D. (2001). Signal transmission in the plant immune response. *Trends in Plant Science*.

[11] Blondelle S. E., Lohner K. (2000). Combinatorial libraries: a tool to design anti-microbial and antifungal peptide analogues having lytic specificities for structure-activity relationship studies. *Biopolymers*.

[12] Bent A. F. (2001). Plant mitogen-activated protein kinase cascades: negative regulatory roles turn out positive. *Proceedings of the National Academy of Sciences of the United States of America*.

[13] Van Loon L. C. (1997). Induced resistance in plants and the role of pathogenesis-related proteins. *European Journal of Plant Pathology*.

[14] Passardi F., Cosio C., Penel C., Dunand C. (2005). Peroxidases have more functions than a Swiss army knife. *Plant Cell Reports*.

[15] Sasaki K., Iwai T., Hiraga S. (2004). Ten rice peroxidases redundantly respond to multiple stresses including infection with rice blast fungus. *Plant and Cell Physiology*.

[16] Lavania M., Chauhan P. S., Chauhan S. V. S., Singh H. B., Nautiyal C. S. (2006). Induction of plant defense enzymes and phenolics by treatment with plant growth-promoting rhizobacteria *Serratia marcescens* NBRI1213. *Current Microbiology*.

[17] Díaz-Vivancos P., Rubio M., Mesonero V. (2006). The apoplastic antioxidant system in Prunus: response to long-term plum pox virus infection. *Journal of Experimental Botany*.

[18] Vera P., Tornero P., Conejero V. (1993). Cloning and expression analysis of a viroid-induced peroxidase from tomato plants. *Molecular Plant-Microbe Interactions*.

[19] Mohammadi M., Kazemi H. (2002). Changes in peroxidase and polyphenol oxidase activities in susceptible and resistant wheat heads inoculated with *Fusarium graminearum* and induced resistance. *Plant Science*.

[20] Chunhua S., Ya D., Bingle X., Xiao L., Yonshu X., Qinguang L. (2001). The purification and spectral properties of PPO I from Nicotianan tababcum. *Plant Molecular Biology*.

[21] Mayer A. M., Harel E. (1979). Polyphenol oxidases in plants. *Phytochemistry*.

[22] Sarvanan T., Bhaskaran R., Muthuswamy M. (2004). Pseudomonas fluorescence induced enzymological changes in banana roots (cv *Rasthali*) against fusarium wilt disease. *Plant Pathology Journal*.

[23] Abeles F. B., Bosshart P., Forrence L. E., Habiz W. (1970). Preparation and purification of glucanase and chitinase from bean leaves. *Plant Physiology*.

[24] Thakker J. N., Shah K., Kothari I. L. (2009). Elicitation, partial purification and antifungal activity of *β*- 1, 3 glucanse from banana plants. *Journal of Pure and Applied Science PRAJNA*.

[25] Roberts W. K., Selitrennikoff C. P. (1988). Plant and Bacteria differ in antifungal activity. *Journal of General Microbiology*.

[26] Mauch F., Staehelin L. A. (1989). Functional implication of the subcellular localization of ethylene-induced chitinase and *β*-1, 3-glucanase in bean leaves. *Plant Cell*.

[27] Ramanathan A., Vidhasekaran P., Samiyappan R. (2000). Induction of defense mechanisms in greengram leaves and suspension-cultured cells by *Macrophomina phaseolina* and its elicitors. *Zeitschrift fur Pflanzenkrankheiten und Pflanzenschutz*.

[28] Anna R. D. C. F. D. A., Dubery I. A. (2000). Panama disease: cell wall reinforcement in banana roots in response to elicitors from *Fusarium oxysporum* f. sp. cubense Race four. *Phytopathology*.

